# Merit and Justice: An Experimental Analysis of Attitude to Inequality

**DOI:** 10.1371/journal.pone.0114512

**Published:** 2014-12-09

**Authors:** Aldo Rustichini, Alexander Vostroknutov

**Affiliations:** 1 Department of Economics, University of Minnesota, Minneapolis, Minnesota, United States of America; 2 Department of Economics, Maastricht University, Maastricht, The Netherlands; George Mason University, United States of America

## Abstract

Merit and justice play a crucial role in ethical theory and political philosophy. Some theories view justice as allocation according to merit; others view justice as based on criteria of its own, and take merit and justice as two independent values. We study experimentally how these views are perceived. In our experiment subjects played two games (both against the computer): a game of skill and a game of luck. After each game they observed the earnings of all the subjects in the session, and thus the differences in outcomes. Each subject could reduce the winnings of one other person at a cost. The majority of the subjects used the option to subtract. The decision to subtract and the amount subtracted depended on whether the game was one of skill or luck, and on the distance between the earnings of the subject and those of others. Everything else being equal, subjects subtracted more in luck than in skill. In skill game, but not in luck, the subtraction becomes more likely, and the amount larger, as the distance increases. The results show that individuals considered favorable outcomes in luck to be undeserved, and thus felt more justified in subtracting. In the skill game instead, they considered more favorable outcomes (their own as well as others') as signal of ability and perhaps effort, which thus deserved merit; hence, they felt less motivated to subtract. However, a larger size of the unfavorable gap from the others increased the unpleasantness of poor performance, which in turn motivated larger subtraction. In conclusion, merit is attributed if and only if effort or skill significantly affect the outcome. An inequality of outcomes is viewed differently depending on whether merit causes the difference or not. Thus, merit and justice are strongly linked in the human perception of social order.

## Introduction

The relationship between merit and justice has been a permanent theme in the philosophical debate. However, no consensus has been reached. At one of the two extremes we have theories of justice and desert which go back to Aristotle and are later developed by John Locke and John Stuart Mill. The common ground for these theories is that a system is just if it allocates rewards according to merit (“Everyone agrees that justice […] must be in accordance with some kind of merit” [1]). At the other extreme, we have conceptual structures like Rawls', which stem from the idea of justice as *fair* allocations, and separate the foundation of justice from the idea of merit. A corollary of this general position is that income and wealth, and the good things in life, should not be distributed according to moral desert [2], [3]. The proof of this corollary is clear: the principle of reward according to merit would not have been chosen in the original position. In presenting this argument, Rawls explicitly reminds us of the dependence of merit on the idea of good, and observes that the lack of agreement on this deprives moral desert of legitimacy: “Having conflicting conceptions of the good, citizens cannot agree on a comprehensive doctrine to specify an idea of moral desert for political purposes” [3]. Moral desert as moral worth of character and actions is what is questioned: Rawls recognizes that previous rules and agreements have to be respected. These agreements produce legitimate claims and earned entitlements, and the expectations produced by these agreements are legitimate. But they are a replacement for moral desert [3].

We propose here to analyze these claims and to test them experimentally. Several hypotheses seem natural, and we review them before we present the experimental design. The first hypothesis concerns the relationship between justice and merit. A key assumption that we are going to test is that social values are well defined and widely accepted, independently of the attitude to merit. First among such values is aversion to inequality. These values are measuring rods of the desirability of social tools, like the incentives provided by rewards for merit. However, if these social values are not defined independently of merit, then such measurement is difficult, and may be inconsistent. For example, suppose that the attitude to inequality in outcomes, its strength and intensity, depends in a crucial way on the perceived merit of these outcomes. Then the two step process (first establish widely-accepted values, then use them to evaluate the desirability of merit criteria) is hard to implement because these criteria are already used in the first step. The question we propose to test experimentally is whether the perception of merit affects attitude to inequality.

A second hypothesis focuses on the nature and motivation behind inequality aversion. Recent studies [4]–[6] have suggested that emotions may have a functional role. Emotions like regret facilitate learning, forcing an individual to consider counterfactual outcomes in the evaluation of his past choices (“What I would have received had I made a different choice”). Recent literature in economics has further developed this theme [7]. An emotion of envy may have a similar functional explanation, forcing us to learn from the fortunes of others to do a better use of our own abilities (“What I would have received had I done what he did” [8]). Such interpretation of social comparison processes was first proposed in social psychology by Festinger [9], [10]. In this view, envy is the social correspondent of regret. But this functional role is meaningful only when the outcomes of others have merit, and meaningless when outcomes are only due to luck. Clearly, there is much more to learn from the success of others when skill and effort are responsible for the outcome, instead of luck. We cannot learn to be lucky, but we can learn to better use our skills if we see others performing better than we do.

A third hypothesis concerns the nature of merit and its recognition. Just as regret and envy are the emotions that underlie a social preference for aversion to inequality, a different emotion may underlie a desire for our own merit to be recognized by others. Recognition of merit is a basic social value, just as aversion to inequality, and, as such, should then be considered a criterion to apply when we measure the success or failure of a society. Adam Smith analyzes this extensively in the chapter “Of the love of praise, and of that of Praiseworthiness” in the *Theory of Moral Sentiments* [11]. People love praise, but “the love of praise seems, at least in a great measure, to be derived from that of praiseworthiness.” The two complement each other: “The love of praise is the desire of obtaining the favorable sentiments of our brethren. The love of praiseworthiness is the desire of rendering ourselves the proper objects of those sentiments.” [TMS, III, 2]. Love of praise is not vanity, precisely because it is aroused under the condition that it has to be considered deserved by he who enjoys it: it is the social recognition of a deserved merit.

We propose to test this basic conceptual assumption experimentally. More specifically, the hypothesis we test is that individuals recognize merit and reveal this in the attitudes they display towards differences in outcomes for which there is ground for merit (either because of the skill required or the effort employed in it). To test this hypothesis, we need a behavioral measurement of this response and two treatments that clearly separate the role of skill and effort on the one hand from the role of luck on the other.

The behavior we measure is the willingness of subjects to reduce inequality in outcomes, which they can do by subtracting money earned by others. This technique was introduced in the experimental literature by Zizzo and Oswald [12] who found that subjects do subtract from others substantially. The separation between merit (here equal, à la Michael Young [13], to the sum of IQ and effort) and luck is achieved by using two tasks in which the outcome is clearly dependent on skill and effort in one case and on pure luck in the other.

Our study is close to several experimental findings in the literature concerning envy and social status. For example, Charness, Masclet and Villeval [14] show experimentally that the ability of workers to sabotage the work of others leads to decreases in overall performance. We observe a similar pattern in our data in subtraction choices after the game of skill. Other studies [15], [16] report that preferences for redistribution vary with socioeconomic characteristics, like race, culture and income level. These studies, like ours, attribute their findings to envy.

## Methods

### Ethics Statement

The protocol for the experiment and the payment was approved by the Institutional Review Board (IRB) of the University of Minnesota (Project title: “Comparison of Task Performance;” IRB Code Number: 0508M72567; PI: Aldo Rustichini). The participants were informed that they were taking part in a research study. Each participant took a seat at a randomly chosen computer terminal. Everyone gave their verbal consent to participating. The verbal consent was approved by the Institutional Review Board.

### Experimental Setup

In the experiment, the subjects played two different games against a computer: a game of skill (*S*) and a game of luck (*L*). The games were described to the subjects as “Hare and Hounds” and “Guessing Game.” The game of skill was a board game. To win against the computer the subject needed to use some logical and analytical skills. In the game of luck, each subject had to guess a number between 0 and 100. The subject won if the chosen number was no more than ten units away from a second number randomly generated by the computer. Thus, the score earned by the subject was entirely determined by chance. Both games were played 10 times in a row. After each 10 repetitions of each game the subjects had the possibility of subtracting money from one of the other participants in the experiment. They could also choose to do nothing. The subjects did not know from whom they were subtracting money.

The experiment had two order treatments: *SL* and *LS*. In the *SL* treatment, the subjects played the game of skill first (10 times), and then had a chance to subtract money. After this, they played the game of luck (10 times) and then again had a chance to subtract money. In the *LS* treatment the order was reversed: first the game of luck was played and then the game of skill (with subtractions after each game). Wealth effects in both treatments were minimal. The average earnings in the two games were $5.53 which is negligible in comparison with the subjects' daily allowance.

### Subjects

All the sessions were conducted at the University of Minnesota. There were five sessions run in the *SL* treatment (three with 16 subjects, one with 14, one with 13). There were 7 sessions run in the *LS* treatment (two with 16 subjects, two with 15, one with 13, one with 10 and 8). All the subjects were undergraduate students taking classes at the University. Each session lasted approximately 45 minutes. Most of the subjects had never participated in economics experiments before.

Before the subjects played the game they were given a verbal presentation of the rules. The subjects could ask questions about the rules of the game at the time of the presentation. The game started once no further questions were asked. The same experimenter presented the rules at all times. The subjects were also instructed to maintain silence and not talk to each other during the play. Experimental instructions can be found in S1 Appendix.

### Materials

The experiment was conducted using *z*-tree [17], LabView 6 and Java based software. The game of skill was realized as a Java applet. This was a modified version of the original applet provided at www.mazeworks.com. The game of luck was realized as part of the *z*-tree program. The data are available at www.vostroknutov.com/envybeh/envybehdata.zip.

### Game of Skill

**Figure 1:**
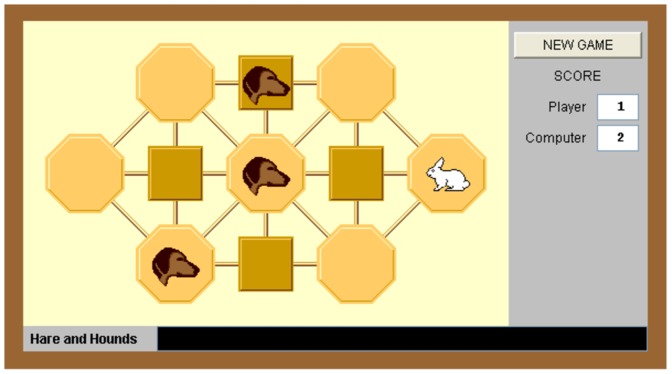
Hare and Hounds game.

The game of skill is the classic *Hare and Hounds* game (see Fig. 1). The subjects played in the role of hounds and the computer played as hare. The hounds have to trap the hare while it is trying to escape. The hare is trapped if no move is feasible when its turn comes. The two players (subject and computer) alternate moves. The subject can choose one and only one hound to move, and he can only move to the right or vertically (up or down) by one cell. The hare can move by one cell in any direction. The hare is declared the winner when it passes to the left of all three hounds, so that capture is impossible. Neither player can choose to move a piece to an occupied position. To move a hound, the subject has to drag and drop it to the cell where she wants to move it. Illegal moves are not allowed by the program. A detailed description of the rules of the game as they were presented to the subjects is reported in S1 Appendix.

The subjects had 20 minutes to play 10 games. They earned $1 for a game won, and nothing for a game lost. The subjects played at different paces: those who completed 10 games earlier than the others were allowed to continue playing without earning any more money. The original program has three levels of difficulty. In the experiment, the level was set to intermediate. The computer selected its moves following an artificial intelligence algorithm as described at www.mazeworks.com.

The Hare and Hounds game is indeed of sufficient complexity for it to be considered a game of skill. Consider the optimal strategy presented in Chapter 21 of Berlekamp, Conway and Guy [18]. In all the trials of the experiment, the initial position was such that the hounds player (in our experiment, the subject) wins if he uses the optimal strategy. This strategy, however, is complex. A way to describe it is to first assign a number between 0 and 3 to every cell, and then classify the position of the four animals on the board according to the sum of these values. The winning strategy is to keep this sum equal to 3 at every move. It is highly unlikely that the subjects understood this strategy and the underlying classification. No evidence of this is given in the debriefing notes at the end of the experiment. Instead, the subjects displayed in their decisions and their statements some understanding of how to avoid the most obvious mistakes, and the ability to look ahead at the hair's next two or three moves. This makes us conclude that the Hare and Hounds game was likely perceived by the subjects as a game of skill for the entire set of ten rounds.

### Game of Luck

The subjects were asked to guess a number between 0 and 100 that was then randomly chosen by the computer (from uniform distribution on [0, 100]). If the subject's guess was within a distance of 10 units from the number chosen by the computer on either side, then the subject earned $1. If it was further than that, the subject earned nothing (see S1 Appendix for the exact instructions). The game was played 10 times.

### Subtraction

**Figure 2:**
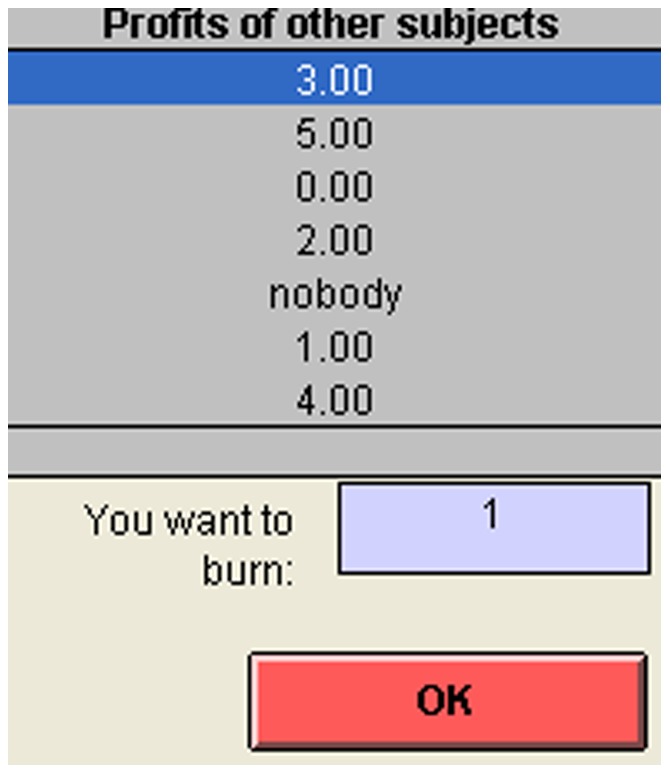
Subtraction screen.

After playing each game 10 times, the subjects were told the amount of money they had won (from $0 to $10). Then, they were offered the choice of subtracting money [12] from another subject, or not (see Fig. 2).

Three possibilities were available. A subject could subtract an amount of money from only one other subject and pay for it. If the subject decided to subtract an amount of *x* dollars from somebody, he had to pay 0.1*x* dollars for it. Both amounts of money would be lost, and not transferred to anybody. Alternatively, he could choose to subtract $1 with probability 0.25 from one other subject and pay nothing. Finally, he could choose to do nothing. A limit on subtractions was imposed so that no subject could lose more than he earned. The subjects were explicitly informed about this point during the oral presentation of the instructions. In addition, the subjects who decided to subtract money and pay for it could not spend more money in payments than they had. The detailed instructions are reported in the S1 Appendix.

The option of probabilistic subtraction with no payment, was introduced to observe the subtraction decisions of the participants who felt the inclination to subtract but were not motivated enough to pay for it. Without this option, the participants with a desire to subtract but without enough motivation would have chosen not to subtract, which would have made them indistinguishable from those participants who genuinely did not want to subtract. The probability of 0.25 (instead of, for example, 0.5) was chosen so that the low probability subtraction option would not become too attractive to those subjects who were willing to pay a small amount to subtract, thus, ensuring that the no payment option was chosen only by those who really did not want to pay to subtract.

It was clear to the subjects that no part of the amount subtracted would be paid to them or to anyone else, which was also emphasized by the use of the word “burn.” This word does not carry any value judgement or suggest one choice over another. It also clearly states that the money subtracted from a participant is destroyed and is not transferred to the person who subtracts. Subtraction was completely anonymous. The subjects could observe a list of the amounts earned by all the other subjects in that session. The amounts were presented in random order. If two subjects won the same amount two entries with that amount were presented. The option of no subtraction was indicated by the item “nobody,” which appeared in a random position in the list. Each subject lost the total amount subtracted in real dollars and the payment for the subtraction if he chose the paying option.

### Treatments

The experiment had two order treatments denoted by *SL* and *LS*. In the first treatment, the steps were in this order: skill game, subtraction phase, display of current earnings, luck game, subtraction phase, and finally display of total earnings for each subject in the experiment. In the *SL* treatment the first time subjects learned about the subtraction phase was after they played the skill game 10 times. The first time they learned about the luck game was after the first subtraction phase. When the luck game instructions were given, the subjects were not told anything about what would happen afterwards, so their behavior was not influenced by the future subtraction phase. In the second treatment, the order was reversed: first the luck game was played, then the subtraction phase took place, then the current winnings were displayed, then the skill game, then the second subtraction, then the display of total earnings.

After the first subtraction phase in both treatments the subjects were shown the amount of money that they had won in the first game. This amount was equal to the sum of the money earned playing the game minus the amount of money that was subtracted from them minus the payments for subtraction. Therefore, the subjects could compute how much money they had lost after the first game. Before the subjects were paid, they were asked to provide written descriptions of how they made their decisions in the experiment. At the end of the experiment, the subjects received a participation payment of $10 plus their winnings after both games net of subtractions and payments for subtractions.

## Results

### Summary of the Data

We start by providing an overview of the data. Table 1 presents some summary statistics by treatment (SL and LS) and game (Skill and Luck): the winnings before and after the subtraction phase; the percentages of subjects who do not subtract/subtract with cost/subtract for free; the mean amounts subtracted and the mean amounts lost due to subtraction.

**Table 1:** Summary statistics of the data by treatment (SL and LS) and game (Skill and Luck).

	Luck (LS)	Skill (LS)	Luck (SL)	Skill (SL)
Number of observations	93	93	75	75
Mean amount won before subtraction ($)	1.85	4.82	2.41	4.93
Mean amount left after subtraction ($)	1.38	4.29	1.61	3.39
Percentage of subjects who did not subtract (%)	32.2	32.2	29.3	34.6
Percentage of subjects who subtracted with cost (%)	22.6	16.1	41.3	42.7
Percentage of subjects who subtracted for free (%)	45.2	51.6	29.3	21.3
Mean amount subtracted ($)	0.75	1.25	1.56	2.49
Mean percentage of winnings paid for subtraction (%)	1.11	1.00	1.23	1.36

Next, we define the variables used in the further analysis and provide some descriptive statistics. In order to analyze the subtraction behavior we need some *unified* measure to “rank” the earnings of the subjects. This is necessary because in each group of subjects the maximum and minimum amounts earned are different and it is likely that, when deciding to subtract, the subjects would consider as reference the maximum and minimum earnings in the group and *not* the absolute maximal and minimal amounts that *could have been earned* (S2 Appendix provides the list of concepts mentioned by the subjects in the end-of-session debriefing: 17.11% of subjects explicitly mention that they chose the person who won the most money in the group as their target). To take this fact into account we introduce the variable Gap, which varies from 0 to 1 and is calculated as follows. Suppose that in a given session after the skill or luck game the maximum amount earned in the group is *M* and the minimum amount earned is *m*. Then, for a subject with earnings *x*, Gap is equal to 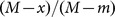. Thus, Gap is a normalized distance of the subject's earnings from the maximum amount earned in the group. Notice that since Gap is just a normalization of the amount earned, it is not correlated with the group size (in the same way the earnings of each subject are independent of the group size (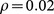)). The variable Gap now allows us to analyze the subtraction decisions of all the subjects from the different sessions. In our analysis we will also use the variable Rank, defined as the complement to 1 of Gap: 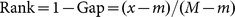. This variable describes the relative position of the subject in the hierarchy of earnings in the group.

**Figure 3:**
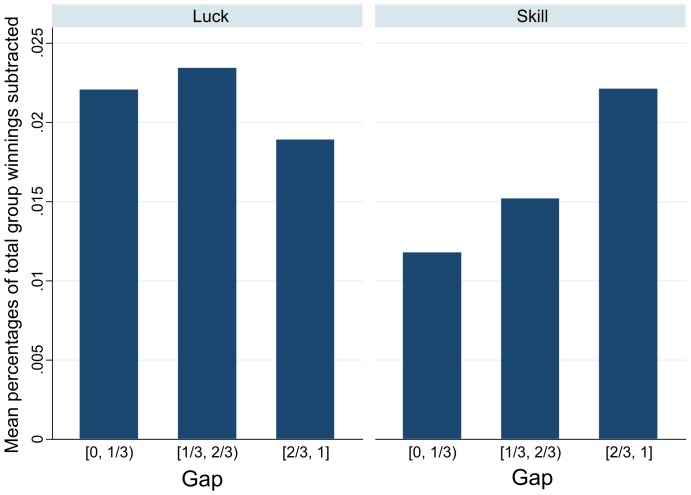
Mean percentages of total group winnings subtracted by subjects with different levels of Gap.

Fig. 3 shows the average percentages of the total group winnings subtracted by subjects with different values of Gap. The rationalization behind using the percentage of *total group winnings* is that the absolute amount subtracted depends on how much is available. This percentage ensures that the groups are comparable. Notice that in the luck game the subjects subtract more than in the skill game overall: the mean percentage subtracted after the luck game is 0.0212% as compared to 0.0168% after the skill game, which is 20% less. However, the nature of the subtraction is different for the two games. After the luck game the subjects with all values of Gap subtract approximately the same amount. After the skill game, subtractions follow a trend: the subjects with low relative earnings (high values of Gap) subtract a lot, while the subjects with high earnings (low Gap) subtract much less.

One potential problem with our design is that the subjects, once they are aware of the subtraction phase, might form expectations about how much others will subtract from them and others. This can lead to some form of reciprocation or other belief-dependent behavior. Reciprocation, for example, is mostly salient for the subjects who are at (or close to) the top: they might reason that the subjects with low earnings will subtract from them, which might lead to them deciding to subtract from the subjects with low earnings in return. In the skill game we observe that the subjects with high earnings subtract much less than those with low earnings. Analysis of the written debriefings shows that *none* of the 168 subjects mentioned this kind of reciprocation or any other behavior which is influenced by beliefs about the others' potential subtraction choices (see S2 Appendix). This strongly suggests that the subjects did not use such reasoning when making subtraction decisions.

The next variable that we look at is the percentage of the winnings of the “target” that the subjects choose to subtract from. To illustrate, if a subject chooses to subtract $4 from a subject who has won $8 then the value of this variable will be 0.5 = 4/8.

**Figure 4:**
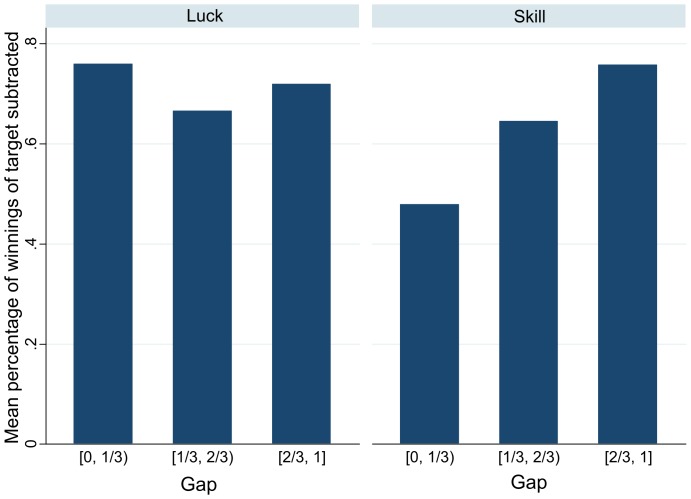
Mean percentages of the winnings of the target subtracted by subjects with different levels of the Gap variable.

Fig. 4 shows the mean percentages of the winnings of the target chosen by subjects with different levels of Gap. The pattern is very similar to that of Fig. 3. After the luck game, all the subjects, regardless of their relative position in the group, choose to subtract the same high percentage of the target's winnings. After the skill game, the situation is different. Subjects with high Gap, or low relative winnings, choose a high percentage of the target's winnings to subtract, while subjects with high relative winnings choose a smaller percentage.

### Decision to Subtract

The subjects choose to subtract frequently: on average, 67.8% of the times. The frequency is similar in the skill games (66.6%) and in the luck games (69.1%). A non-parametric test shows that the difference between the two is not significant (two-sample Wilcoxon-Mann-Whitney rank-sum test, *z* = 0.467, *p* = 0.64). However, if we consider the effect of the social ranking, measured by the difference between the amount earned by the subject and the maximum amount earned in the group, the picture changes completely.

Table 2 reports the logit estimate of the probability that a subject subtracts money from someone else (S1 Table reports similar regression with the linear probability model). The dependent variable is equal to 1 if the subject decides to subtract money from others, either with the paying option or with the zero-cost option. The independent variables are Gap, the type of game (Skill or Luck) and the interaction between these two variables. The variable Skill is equal to 1 if the game is a game of skill, and 0 if the game is a game of Luck. Standard errors are adjusted for clusters in the identity of the session, the top level nested cluster [19].

**Table 2:** Decision to subtract: logit clustered by session.

	1	2	3	4	5	6
	All obs.	All obs.	All obs.	First Game	Pay	No Pay
	b/se	b/se	b/se	b/se	b/se	b/se
Gap	0.567	0.566	−0.519	−0.964	−0.889	−0.284
	(0.458)	(0.459)	(0.566)	(0.908)	(0.619)	(0.591)
Skill		−0.108	−1.265***	−1.885**	−1.316**	−1.315**
		(0.211)	(0.482)	(0.955)	(0.596)	(0.585)
Gap × Skill			2.318***	3.522***	2.351**	2.502***
			(0.626)	(1.227)	(1.034)	(0.605)
constant	0.459	0.514	1.078**	1.298**	0.460	0.361
	(0.362)	(0.356)	(0.451)	(0.657)	(0.492)	(0.457)
N	336	336	336	168	208	237

All models are logit. Models 1, 2 and 3 are estimated over all observations. Model 4 is estimated only on those observations in which the subjects played the first game of the session. In model 5, the dependent variable is only subtraction with payment (or choice of “nobody”); in model 6, only subtraction without payment (or choice “nobody”). S4 Table contains similar regressions but with the absolute amounts won instead of Gap.

Consider model 3 in Table 2. The Gap variable has no direct significant effect, but has a significant effect when interacted with Skill. Everything else being equal, the subjects subtract less in a skill game: the coefficient of the variable Skill is negative and significant (*z* = −2.62, *p* = 0.009). However, the response to the Gap variable is stronger in the Skill game than in the Luck game: the coefficient of the interaction between the two is positive and significant (*z* = 3.70, *p* = 0.000). The results are virtually unchanged if we restrict the same analysis to observations where the subjects are playing the first game only (the skill game in the SL treatment and the luck game in the LS treatment) and have no previous experience with the subtraction decision of others (model 4). The logit regression restricted to these observations has coefficient −1.88 (*z* = −1.970, *p* = 0.049) for the Skill variable and coefficient 3.52 (*z* = 2.87, *p* = 0.004) for the interaction term.

To test whether there is a difference in behavior for the two subtraction methods we ran the same regression as model 3 but excluding either non-paying subtractors (model 5) or paying subtractors (model 6). Thus, model 5 has only observations of non-subtractors and subtractors who paid and model 6 has only observations of non-subtractors and subtractors who did not pay. The results are quantitatively the same: the sign and size of the coefficients are similar to those in model 3. The variables Skill and the interaction between Gap and Skill are still significant. This shows that the two subtraction methods in our experiment produce the same behavior.

The size of the interaction effect between the two variables Gap and Skill on the probability of the subject subtracting money from another subject is estimated in Table 3. Since the model is non-linear (with a logit specification), we use the estimation of the effect size and significance with the method described in Ai and Norton [20] and Norton, Wang, and Ai [21]. The interaction, in a non-linear model, varies with the values of the two variables.

**Table 3:** Interaction effect.

variable	Mean	Standard Deviation	Minimum	Maximum
interaction effect	0.484	0.061	0.369	0.551
standard error	0.155	0.015	0.131	0.177
*z* value	3.098	0.178	2.808	3.478
interaction effect	0.710	0.103	0.508	0.823
standard error	0.227	0.036	0.170	0.278
*z* value	3.162	0.506	2.632	4.390

Cross partial derivative of Gap and Skill on the probability of subtracting (see [21] for a presentation of the method) in the logit model 3 (first three rows) and model 4 (last three rows) of Table 2.

**Figure 5:**
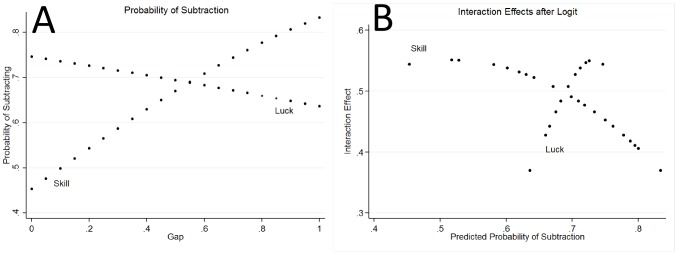
Probability of subtraction and interaction effect. (A) Estimated probability of subtraction. On the horizontal axis: Gap. On the vertical axis: probability of subtracting. (B) Estimate of the interaction effect. The interaction effect is between Gap and Skill; the underlying model is a logit. On the horizontal axis: probability of subtracting; on the vertical axis: delta method estimate of the interaction effect. In Luck games the probability is close to a constant function of the game variable, around the mean value (of 69.1%). In the skill game, the probability of subtracting varies with the Gap variable, and covers a large range.

The interaction effect is large: the Gap variable adds an average of 48% (or 71% when we only consider the first game) to the probability of subtracting in the skill game over the range of its value, while in luck games the effect is statistically zero. In other words, the probability of subtracting is approximately constant with respect to Gap in the luck games. Instead, in skill games the probability increases from values lower than in the luck games when the gap is small, to values that are higher when the gap is large. The two curves cross, since the average probability is, as we have just seen, the same in the two games. The crossing is illustrated in the left-hand panel of Fig. 5.

The size of the interaction effect is different in the skill and luck games. This is reported in the right-hand panel of Fig. 5. Note that on the horizontal axis we report the probability of subtraction. The figure clearly displays two branches: a steep increasing one for the luck games, and a flatter, decreasing one for the skill games. The reason for this pattern is clear. As we have seen, the probability of subtraction in luck games is almost constant around its mean rate, so the corresponding branch in the estimate of interaction effects is almost vertical.

Finally, an estimate of the logit regression of the Gap variable, separately for the Skill and Luck games, has a direct interpretation. In the Luck regression, the marginal effect of Gap is statistically zero (marginal effect  = −0.11, *z* = −0.99, *p* = 0.322). In the Skill games the marginal effect is significant, positive and large (marginal effect  = 0.393, *z* = 3.17, *p* = 0.002).

### Amount Subtracted

So far, we have considered the decision to subtract or not. We now consider how the amount subtracted depends on the Gap and Skill variables. We recall that a subject could subtract an amount of *x* dollars from any other by paying 0.1*x* dollars for it. Both amounts of money would be lost and not transferred to anybody. The subjects also had the option of subtracting 1 dollar with probability 25%, at no cost. We look at the variable called Amount Subtracted, which is equal to 0 if the subject chose not to subtract, equal to the chosen amount for a paying subtraction; and equal to 0.25 for the no-cost subtraction. In the last case 0.25 corresponds to the *expected* amount subtracted. The results are reported in Table 4.

**Table 4:** Amount Subtracted: OLS clustered by session.

	1	2	3	4
	All obs.	All obs.	All obs.	First Game
	b/se	b/se	b/se	b/se
Gap	0.418	0.423	−0.148	−0.068
	(0.270)	(0.254)	(0.223)	(0.272)
Skill		0.428*	−0.200	−0.162
		(0.196)	(0.310)	(0.402)
Gap × Skill			1.213	2.517***
			(0.699)	(0.602)
constant	0.767***	0.551***	0.848***	0.543**
	(0.142)	(0.125)	(0.169)	(0.195)
				
N	336	336	336	168

The dependent variable is the amount subtracted. To take into account the amount subtracted by subjects who did not pay for subtraction, we impute in these cases an amount subtracted equal to the expected value subtracted (0.25×$1 = 25 cents). S4 Table contains a similar regression but with absolute amounts won instead of Gap.

The 4th column model, estimated on only first game observations, has good significance (*F*(3, 11) = 10.81, *p*<0.0013). Subjects subtract around $2.5 more per unit of Gap after the Skill game (*t* = 4.18, *p* = 0.002). After the Luck game, Gap has no significant effect on the amounts subtracted. The variable Skill has a negative coefficient, as it had in the decision to subtract, but is not significant. S2 Table reports the regression of the amount subtracted only by subjects who are willing to subtract at a cost. In this case, we do not change the variable Amount Subtracted by putting the expected amount 0.25 for those who chose no-cost subtraction and just leave it at 0 for these subjects. The results are qualitatively the same: the model for the first game is significant (F(3, 11) = 10.64, *p*<0.0014). Around $2.4 more is subtracted per unit of Gap after the Skill game (*t* = 3.97, *p* = 0.002). After the game of Luck, Gap plays no significant role in the decision on the amount to subtract.

If we look at all the observations (model 3), the results are qualitatively the same: only the interaction of Gap and Skill is borderline significant. The change in the amount subtracted is $1.2 per unit of Gap after the Skill game (*t* = 1.74, *p* = 0.111) and does not depend significantly on Gap after the Luck game. This shows that the nature of the game played (Skill or Luck) has the same effect on the amounts subtracted regardless of the order.

### Amount Lost

The total amount subtracted does not take into account the amount earned by the subject from whom the money is subtracted. We will refer to this latter subject as the “target.” We examine now whether the percentage of the winnings subtracted from the target is affected by the nature of the game. For the non-paying subtractors we look at the expected percentage of winnings subtracted by taking 25 cents as the expected amount subtracted. The results are reported in Table 5.

**Table 5:** Fraction of the amount subtracted over the amount earned by the target, including subtraction at no cost: OLS clustered by session.

	1	2	3	4
	All obs.	All obs.	All obs.	First Game
	b/se	b/se	b/se	b/se
Gap	0.022	0.022	−0.110**	−0.092
	(0.040)	(0.042)	(0.048)	(0.067)
Skill		−0.056*	−0.201***	−0.148*
		(0.028)	(0.062)	(0.079)
Gap × Skill			0.279	0.489***
			(0.132)	(0.133)
constant	0.217***	0.245***	0.313***	0.237***
	(0.035)	(0.039)	(0.050)	(0.041)
				
N	336	336	336	168

The dependent variable is the ratio of the amount subtracted and the amount won by the target. S4 Table contains a similar regression but with absolute amounts won instead of Gap.

In the model restricted to the observations from the first games only (model 4), the interaction of Gap and Skill is significant and positive: subjects with Gap equal to 1, or those who won the least number of games of Skill, choose to subtract 48.9% more from their targets' winnings than subjects with Gap equal to 0 (*t* = 3.66, *p* = 0.004). As the subjects decide the percentage of winnings to subtract they are sensitive to the distance of their outcome from the top result when the game is a game of Skill but not if it is of Luck. Thus, the outcome after the Skill game is a signal of some hidden important quality (S3 Table reports similar regressions for the fraction of winnings subtracted, but treating the no-cost subtractors as non-subtractors: for the first game model only the interaction of Gap and Skill is significant, with an increase of 45.8% in the fraction subtracted per unit of Gap after the Skill game (*t* = 3.35, *p* = 0.006) and no significant increase after Luck). Skill variable is also marginally significant: after the Skill game the subjects overall subtract 15% less of their targets' winnings than after the Luck game (*t* = −3.66, *p* = 0.004). However, after the Skill game the subjects also increase their percentages as their Gap grows. This is exactly in line with Fig. 4, which reports the mean amounts of the percentages subtracted. This points towards our hypothesis that subtraction after the Luck game is more justifiable (thus there is more subtraction overall), but subtraction after the Skill game depends on merit (and thus dependency on Gap).

### Choice of Target

The subjects could choose one of the other participants in the session as the target for subtraction. The only information they had to differentiate among them was the amount they had earned. Who among the other participants was the favorite target? Approximately half of the times, the subjects who subtracted chose the top winner in the game. This is true for both games (49 times out of 112 in the skill game, and 50 times out of 116 in the luck game.) To estimate more precisely what was affecting the choice of the target, we test a general hypothesis that subtraction is influenced by the rank of the target (defined as 1 – Gap) and the nature of the environment (Skill or Luck). Thus, we look at each subject as a potential victim, and consider the total Amount Lost by the subject, which is equal to the sum of the subtractions made by all the other subjects in the session (which can be potentially higher than the amount of money earned by the subject). The results are reported in Table 6.

**Table 6:** Amount lost: OLS clustered by session.

	1	2	3	4
	All obs.	All obs.	All obs.	First Game
	b/se	b/se	b/se	b/se
Rank Target	1.927**	1.927**	1.800**	1.670***
	(0.681)	(0.681)	(0.700)	(0.529)
Skill		−0.009	−0.140	−0.339
		(0.080)	(0.360)	(0.320)
Rank Target × Skill			0.271	2.742**
			(0.720)	(1.175)
constant	0.056	0.061	0.122	−0.238
	(0.176)	(0.208)	(0.286)	(0.172)
				
N	336	336	336	168

The dependent variable is the total amount lost by the target, obtained by adding the amounts subtracted by all the subjects choosing him as target. The variable Rank Target takes values in the unit interval, and is the complement to 1 of the Gap variable.

As predicted, the variable Rank Target is significant and positive (model 3), which supports the hypothesis that subjects at the top of the performance scale are favorite targets for subtraction. The interaction in model 3, which contains all the observations is not significant. However, if we look at model 4, which has only first game observations, we see a completely different picture. The interaction is significant and 1.5 times larger than the coefficient on Rank Target. This model shows that in the first game subjects with Rank equal to 0 are not targeted at all in either the Skill or Luck games (constant and Skill are insignificant). Subtraction from subjects with Rank equal to 1 is $1.67 on average after the Luck game and $4.41 for subjects with Rank equal to 1 after the Skill game. These amounts are high: the average highest winning in the Luck game is $4.17 and the average highest winning in Skill game is $8.00. Therefore, on average the top subjects lose roughly half of their earnings according to this model.

## Discussion

The experimental test we have run has yielded several main results, which shed light on how individuals perceive the nature of justice, moral desert, and the connection between them.

First, when individuals evaluate differences in outcomes they take into account the origin of the inequality, what caused it or affected it, and adjust their evaluation accordingly. A difference in earnings entirely due to luck is regarded in a completely different way from one due to a combination of skill and effort. Individuals attach merit to an outcome when it is due to skill, and do not when it is due to luck. Thus, the concepts of moral desert and justice are deeply connected, and one needs the other for a proper definition.

Second, when individuals evaluate policies providing a remedy to inequality, they have a mixed attitude. We may see two principles in action that explain their behavior, which can be clearly seen in the way in which subtraction behavior depends on the Gap in Fig. 5. The first is the merit principle: personal responsibility for an outcome is the basis for merit. In the classical conceptualization of Kleinig [22], moral desert is a triadic property, linking a deserving subject, a deserved object, and a basis, in virtue of which the object is deserved by the subject. A necessary condition for a basis of desert is usually taken to be the personal responsibility of the subject: A subject deserves an object in virtue of some fact or event only if the subject is responsible for that fact [23]–[25]. Individuals do not deserve what comes to them without responsibility, for example if it is entirely due to chance. In the latter case, it is acceptable to subtract earnings from others (for example, in the form of taxes, or, in our experiment, through direct subtraction). This principle, however, comes into conflict with the signaling principle: a superior performance in a game that involves skill and effort is a signal of superior ability, while it is not when the task is only based on chance [26]–[30]. Hence, a better performance in a skill task by someone else has a stronger, and negative, affective impact on the individual who observes that superior performance.

The interaction of these two principles explains the pattern of behavior observed at the moment of subtraction. If an outcome is due to chance and luck, then reduction of inequality is justified. In fact, everything else being equal, individuals subtract more in luck than in skill games. If the outcome is due to skill, then the negative impact of the gap between the performance of others and that of the individual who observes it is proportional to the size of the gap, which is what we observe.

A final result provides support for the hypothesis that emotions responding to comparisons of outcomes among peers have a functional reason. Our results show that individuals are more sensitive to differences due to skill than to luck, as they should be because the first is a useful signal of permanent characteristics of an individual and the second is not. This suggests a final concluding comment. Reducing inequality makes an important social signal less reliable, which is a possible functional role of redistribution. From this point of view, the notion of *Homo Faber*, cited at the beginning, acquires an important psychological significance. The notion, as it was originally developed, signified a recognition of the fact that human skill and effort, or merit, was the principal source of human accomplishments, instead of Luck. Claiming the merit for human accomplishments away from the hands of *Fortuna* was also a way to claim that human actions should be guided on the basis of moral desert. *Homo Faber* is also the ethical principle of giving to each what he deserves, not just a descriptive statement.

## Supporting Information

S1 Table **Decision to subtract with linear probability model: OLS clustered by session.**
(PDF)

S2 Table **Amount Subtracted after payment: OLS clustered by session.** The dependent variable is the amount subtracted by the subjects who paid for the subtraction.
(PDF)

S3 Table **Fraction of amount subtracted over amount earned by the target: OLS clustered by session.**
(PDF)

S4 Table **Regressions with absolute amounts.** Three regressions as presented in the main text but with the absolute amount won (Won) instead of the Gap variable. All regressions are run on the data from the first games only.
(PDF)

S1 Appendix **Experimental Instructions.**
(PDF)

S2 Appendix **Debriefing results.**
(PDF)

## References

[1] Aristotle (2000) Nicomachean Ethics. Cambridge, UK: Cambridge University Press.

[2] Rawls J (2001) Justice as fairness: a restatement. Cambridge, MA: Belknap Press, Harvard University.

[3] Rawls J (1971) A Theory of Justice. Cambridge, MA: Harvard University Press.

[4] Camille N, Coricelli G, Sallet J, Pradat-Diehl P, Duhamel JR, et al (2004) The involvement of the orbitofrontal cortex in the experience of regret. Sci 304:1167–1170. 10.1126/science.1094550 15155951

[5] Coricelli G, Critchley HD, Joffily M, O′Doherty JP, Sirigu A, et al (2005) Regret and its avoidance: a neuroimaging study of choice behavior. Nat Neurosci 8:1255–1262. 16116457 10.1038/nn1514

[6] Coricelli G, Dolan RJ, Sirigu A (2007) Brain, emotion and decision making: the paradigmatic example of regret. Trends Cogn Sci 11:258–265. 17475537 10.1016/j.tics.2007.04.003

[7] Rayo L, Becker GS (2007) Evolutionary efficiency and happiness. J Polit Econ 115:302–37.

[8] Bault N, Coricelli G, Rustichini A (2008) Interdependent utilities: how social ranking affects choice behavior. PLoS One 3:e3477. 18941538 10.1371/journal.pone.0003477 PMC2568945

[9] Festinger L (1954) A theory of social comparison processes. Hum Relat 7:117–140.

[10] Festinger L (1954) Motivation leading to social behavior. In: Jones MReditor. Nebraska Symposium on Motivation. Lincoln: University of Nevada Press.

[11] Smith A (1759) Theory of Moral Sentiments. Boston: Wells and Lilly.

[12] Zizzo DJ, Oswald AJ (2001) Are people willing to pay to reduce others' incomes? Ann Econ Stat (1986) 63:39–62.

[13] Young M (1958) The Rise of Meritocracy, 1870–2033. London: Thames and Hudson.

[14] Charness G, Masclet D, Villeval MC (2013) The dark side of competition for status. Manage Sci 60(1):38–55.

[15] Beckman SR, Formby JP, Smith WJ, Zheng B (2002) Envy, malice and Pareto efficiency: an experimental examination. Soc Choice Welfare 19:349–367.

[16] Beckman SR, Zheng B (2007) The Effects of race, income, mobility and political beliefs on support for redistribution. Res Econ Inequal 14:363–385.

[17] Fischbacher U (2007) z-Tree: Zurich toolbox for ready-made economic experiments. Exper Econ 10(20):171–178.

[18] Berlekamp ER, Conway JH, Guy RK (2003) Winning ways for your mathematical plays. Natick, MA: A.K. Peters Ltd.

[19] Cameron AC, Gelbach JB, Miller DL (2011) Robust Inference With Multiway Clustering. J Bus Econ Stat 29(2):238–249.

[20] Ai CR, Norton EC (2003) Interaction term in logit and probit models. Econ Lett 80(1):123–129.

[21] Norton EC, Wang H, Ai C (2004) Computing interaction effects and standard errors in logit and probit models. Stata J 4(2):154–167.

[22] Kleing J (1971) The Concept of desert. Am Philos Q 8:71–78.

[23] Cupit G (1996) Desert and responsibility. Can J Philos 26:83–100.

[24] Feldman F (1996) Responsibility as a condition for desert. Mind 105:165–168.

[25] Miller D (1996) Two cheers for meritocracy. J Polit Philos 4:277–301.

[26] Zahavi A (1975) Mate selection - a selection for a handicap. J Theor Biol 53:205–214. 1195756 10.1016/0022-5193(75)90111-3

[27] Zahavi A (1977) The cost of honesty (further remarks on the handicap principle). J Theor Biol 67:603–605. 904334 10.1016/0022-5193(77)90061-3

[28] Zahavi A, Zahavi A (1997) The handicap principle: a missing piece of Darwin's puzzle. Oxford, UK: Oxford University Press.

[29] Grafen A (1990) Biological signals as handicaps. J Theor Biol 144:517–546. 2402153 10.1016/s0022-5193(05)80088-8

[30] Spence AM (1973) Job market signaling. Q J Econ 87:355–374.

