# Correction: Merit and Justice: An Experimental Analysis of Attitude to Inequality

**DOI:** 10.1371/journal.pone.0117646

**Published:** 2015-02-23

**Authors:** The PLOS ONE Staff

The competing interests statement is incorrect; the publisher apologizes for the error. The correct statement is: The authors confirm that co-author Aldo Rustichini is an academic editor of PLOS ONE. This does not alter the authors’ adherence to PLOS ONE Editorial policies and criteria.

## References

[1] Rustichini A, Vostroknutov A (2014) Merit and Justice: An Experimental Analysis of Attitude to Inequality. PLoS ONE 9(12): e114512 doi: 10.1371/journal.pone.0114512 25490094 10.1371/journal.pone.0114512 PMC4260855

