# Supplementary material for: Merit and Justice: An Experimental Analysis of Attitude to Inequality
Source: PLoS One. 2014 Dec 9;9(12):e114512. doi: 10.1371/journal.pone.0114512 (PMC4260855; doi:10.1371/journal.pone.0114512)
Supplement: S1 Table — Decision to subtract with linear probability model: OLS clustered by session. (PDF) [file pone.0114512.s001.pdf]

Supporting Information for the article

“Merit and Justice: An Experimental Analysis of Attitude to Inequality”

by Aldo Rustichini and Alexander Vostroknutov

Table S1

Decision to subtract: OLS clustered by session.

	1	2	3	4	5	6
	All obs.	All obs.	All obs.	First Game	Pay	No Pay
	b/se	b/se	b/se	b/se	b/se	b/se
Gap	0.123	0.123	–0.110	–0.204	–0.220	–0.070
	(0.103)	(0.103)	(0.115)	(0.176)	(0.150)	(0.146)
Skill		–0.023	–0.280**	–0.408*	–0.319**	–0.312**
		(0.046)	(0.106)	(0.205)	(0.139)	(0.134)
Gap × Skill			0.495***	0.734**	0.571**	0.587***
			(0.132)	(0.250)	(0.240)	(0.134)
constant	0.615***	0.626***	0.748***	0.791***	0.614***	0.590***
	(0.084)	(0.082)	(0.090)	(0.123)	(0.120)	(0.112)
N	336	336	336	168	208	237
