# Supplementary material for: Merit and Justice: An Experimental Analysis of Attitude to Inequality
Source: PLoS One. 2014 Dec 9;9(12):e114512. doi: 10.1371/journal.pone.0114512 (PMC4260855; doi:10.1371/journal.pone.0114512)
Supplement: S2 Table — Amount Subtracted after payment: OLS clustered by session. The dependent variable is the amount subtracted by the subjects who paid for the subtraction. (PDF) [file pone.0114512.s002.pdf]

Supporting Information for the article

“Merit and Justice: An Experimental Analysis of Attitude to Inequality”

by Aldo Rustichini and Alexander Vostroknutov

Table S2

Amount Subtracted after payment: OLS clustered by session. The dependent variable is the amount subtracted by the subjects who paid for the subtraction.

	1	2	3	4
	All subs.	All subs.	All subs.	First Game
	b/se	b/se	b/se	b/se
Gap	0.384	0.389	–0.152	–0.036
	(0.271)	(0.253)	(0.223)	(0.276)
Skill		0.428*	–0.167	–0.059
		(0.202)	(0.307)	(0.392)
Gap × Skill			1.149	2.435***
			(0.718)	(0.614)
constant	0.690***	0.473***	0.755***	0.413*
	(0.139)	(0.125)	(0.171)	(0.196)
N	336	336	336	168
