# Supplementary material for: Merit and Justice: An Experimental Analysis of Attitude to Inequality
Source: PLoS One. 2014 Dec 9;9(12):e114512. doi: 10.1371/journal.pone.0114512 (PMC4260855; doi:10.1371/journal.pone.0114512)
Supplement: S3 Table — Fraction of amount subtracted over amount earned by the target: OLS clustered by session. (PDF) [file pone.0114512.s003.pdf]

Supporting Information for the article
"Merit and Justice: An Experimental Analysis of Attitude to Inequality"
by Aldo Rustichini and Alexander Vostroknutov

Table S3

Fraction of amount subtracted over amount earned by the target: OLS clustered by session.

	1	2	3	4
	All subs.	All subs.	All subs.	First Game
	b/se	b/se	b/se	b/se
Gap	0.014	0.014	–0.115**	–0.073
	(0.039)	(0.041)	(0.051)	(0.070)
Skill		–0.037	–0.179**	–0.096
		(0.028)	(0.064)	(0.077)
Gap × Skill			0.274*	0.458***
			(0.138)	(0.137)
constant	0.192***	0.210***	0.278***	0.180***
	(0.035)	(0.039)	(0.052)	(0.041)
N	336	336	336	168
