# Supplementary material for: Merit and Justice: An Experimental Analysis of Attitude to Inequality
Source: PLoS One. 2014 Dec 9;9(12):e114512. doi: 10.1371/journal.pone.0114512 (PMC4260855; doi:10.1371/journal.pone.0114512)
Supplement: S4 Table — Regressions with absolute amounts. Three regressions as presented in the main text but with the absolute amount won (Won) instead of the Gap variable. All regressions are run on the data from the first games only. (PDF) [file pone.0114512.s004.pdf]

Supporting Information for the article
“Merit and Justice: An Experimental Analysis of Attitude to Inequality”
by Aldo Rustichini and Alexander Vostroknutov

Table S4

Three regressions as presented in the main text but with the absolute amount won (Won) instead of the Gap variable. All regressions are run on the data from the first games only.

	1<br>Decision to Subtract<br>b/se	2<br>Amount Subtracted<br>b/se	3<br>Fraction Subtracted<br>b/se
Won	0.031	0.025	0.025*
	(0.034)	(0.057)	(0.014)
Skill	0.342***	2.866***	0.498***
	(0.050)	(0.311)	(0.063)
Won × Skill	–0.093***	–0.370***	–0.095***
	(0.029)	(0.078)	(0.017)
constant	0.626***	0.458***	0.139***
	(0.057)	(0.146)	(0.031)
N	168	168	168
