# Supplementary material for: Merit and Justice: An Experimental Analysis of Attitude to Inequality
Source: PLoS One. 2014 Dec 9;9(12):e114512. doi: 10.1371/journal.pone.0114512 (PMC4260855; doi:10.1371/journal.pone.0114512)
Supplement: S1 Appendix — Experimental Instructions. (PDF) [file pone.0114512.s005.pdf]

Supporting Information for the article
“Merit and Justice: An Experimental Analysis of Attitude to Inequality”
by Aldo Rustichini and Alexander Vostroknutov

Appendix S1. Experimental Setup

Rules of the Game

This is the description of the rules taken from www.mazeworks.com:

“On each turn, a single hound moves to a directly connected empty square or octagon, followed by the hare’s similar move. The hounds may only move vertically or forward (to the right), not backward. The hare may move in any direction. To move a piece, drag and drop it with the mouse. There are no captures. The hounds win by trapping the hare so that he is unable to move. The hare can win in two ways. He can escape, which he does by moving past (to the left of) all three hounds. Also, if the hounds move 10 times in a row without advancing (i.e. they only move up and down) then the hounds are stalling and the hare wins.”

These are the instructions given to the subjects on the screens while they were choosing: “In each period, a random number X between 0 and 100 is generated. Please choose a number Z between 0 and 100. Your profit in this period is $1 if the distance between Z and X is less than 10 and $0 otherwise.”

Instructions for subtraction

The following instructions were presented to subjects on their screens

“Here you see the earnings of all subjects but yourself. You have a possibility to subtract money from somebody else. You can do one of three things: 1) Choose a subject from the list and enter a positive amount to subtract; 2) Choose a subject from the list and enter zero to subtract; 3) Choose “nobody” from the list and enter zero to subtract. In case 1, you have to pay for subtracting money: if you choose to subtract Y dollars, you lose 0.1 Y dollars. In case 2, $1 will be subtracted from the chosen subject with probability 25% and you don’t pay anything. In case 3, nothing happens. Your earnings are now $4. Therefore, in case 1 you can choose to subtract up to $40 dollars.”[1]

Debriefing

After the experiment, the subjects where asked the following question: “Can you please comment on how you made the decisions in this experiment?” They could write anything they wanted in the text box.

[1]The instructions were presented verbally using slides. The subjects were told during the presentation that they cannot lose more money than they earned through subtractions.
