# Supplementary material for: Merit and Justice: An Experimental Analysis of Attitude to Inequality
Source: PLoS One. 2014 Dec 9;9(12):e114512. doi: 10.1371/journal.pone.0114512 (PMC4260855; doi:10.1371/journal.pone.0114512)
Supplement: S2 Appendix — Debriefing results. (PDF) [file pone.0114512.s006.pdf]

Supporting Information for the article
"Merit and Justice: An Experimental Analysis of Attitude to Inequality"
by Aldo Rustichini and Alexander Vostroknutov

Appendix S2

After the experiment, the subjects commented on how they made their decisions. The following table summarizes the answers regarding the subtraction choices. The first two columns contain a description of the type of answer. The last column shows the percentage of subjects who gave such an answer.

**Alias**	**Description**	**Percentage**
Just games	Subjects did not mention at all the subtraction part of the experiment	20.39%
No point	"There is no point in burning anyone's money"	19.08%
Top	"I burned money from the person who earned the most"	17.11%
No pay	Subjects mentioned choosing the no pay subtraction option	16.45%
Tit-for-tat	"I did not burn any money because I thought others won't burn anything from me"	13.82%
Random	Subjects mentioned choosing a random target to burn money from	12.50%
No gain	"I did not burn any money because there was nothing to gain"	9.21%
No idea	"I did not understand the purpose of burning money"	7.24%
No hurt	"I did not burn any money because I did not want to hurt anyone"	6.58%
Revenge	"I burned money because someone burned money from me before"	5.26%
Middle	Subjects mentioned burning money from someone in the middle (not top)	2.63%
Equality	Subjects mentioned burning money to equalize the winnings	1.97%
Fun	Subjects mentioned burning money because it is "fun"	1.97%
Think top	Subject mentioned that they think others will burn money from the person with the highest winnings	1.31%
None	Subjects left the text box blank	2.63%

Summary of the answers regarding the subtraction choices. The percentages do not sum up to 100% since some subjects mentioned several features in their answers.
